# The impact of heart irradiation dose on cardiac injury and survival in lung cancer patients after radiotherapy

**DOI:** 10.3389/fonc.2025.1675772

**Published:** 2025-09-25

**Authors:** Bingchen Li

**Affiliations:** Southeast University, Nanjing, China

**Keywords:** lung cancer, radiotherapy, heart irradiation dose, survival, RIHD

## Abstract

Thoracic radiotherapy is a primary treatment modality for lung cancer, with approximately two-thirds of patients receiving it. The association between heart dose and post-radiotherapy survival and cardiac injury represents a critical area of contemporary radiotherapy research, yet understanding of radiation-induced heart disease (RIHD) in lung cancer remains incomplete. This review synthesizes literature on the effects of heart dose on survival and substructure-specific cardiac injury in lung cancer patients, evaluating thresholds for reversible and irreversible damage to cardiac substructures. We further summarize key mechanisms underlying RIHD.

## 1 Introduction

Lung cancer is a prevalent type of cancer with a increasing incidence and mortality. According to statistical analysis of data from the American Society of Clinical Oncology (ASCO), in 2020, lung cancer was the second most commonly diagnosed cancer in both men and women in the United States, but it was the leading cause of cancer-related deaths among both genders, with a mortality rate of 23% in men and 22% in women (1). Early-stage lung cancer is often asymptomatic, and approximately one-third of patients have already progressed to the locally advanced stage by the time they present with symptoms, which can result in the loss of the opportunity for surgical treatment (2). Radical surgery may not be a viable option for some early-stage patients due to poor general health or other reasons. For the past 30 years, radical concurrent chemoradiotherapy has been the standard treatment for locally advanced unresectable non-small cell lung cancer (3). However, the time to disease progression after chemoradiotherapy is only approximately 8 months, with a 5-year survival rate of less than 15% (2, 4). The Radiation Therapy Oncology Group (RTOG) 7301 study established that a radiation dose of 60–63 Gy (single dose of 1.8-2.0 Gy) used to treat non-small cell lung cancer (5). Subsequently, studies have attempted to increase the radiation dose to improve survival outcomes (6–10). Published in 2015, RTOG 0617 is a phase III randomized controlled clinical trial that employed a dose-escalating radiotherapy design for the treatment of stage III unresectable lung cancer (11). The study demonstrated that contrary to mainstream review, increasing the radiation dose did not result in improved survival benefits (11). The secondary analysis of RTOG 0617 revealed that the survival of patients with locally advanced non-small cell lung cancer is associated with the radiation dose received by the heart (12).

## 2 Methods

### 2.1 Search strategy

PubMed, Embase, Cochrane Library, and Web of Science databases were searched. The time range of the literature was from 2010 to 2025 in each database, and the language was limited. The medical subject terms used were as follows: lung cancer, NSCLC, radiotherapy, radiation therapy, cardiac toxicity, heart dose, RIHD.

### 2.2 Inclusion criteria

(1) Subjects: patients with pathologically confirmed lung cancer (NSCLC or SCLC); (2) Interventions: radiotherapy; (3) Study types: retrospective/prospective; (4) Outcome indicators: overall survival (OS), progression-free survival (PFS), pericarditis, myocardial infarction, heart failure, arrhythmia, etc.

### 2.3 Exclusion criteria

Articles with the following conditions will be excluded: (1) animal or cell experiments, case reports, scientific experiment plans, reviews, letters, editorials, conference papers, etc.; (2) articles with missing data or serious errors; (3) repeated publications; (4) no data on survival or cardiac events were reported; (5) The full text was not found.

### 2.4 Data extraction

The retrieved literature was imported into Zotero, and the title and abstract of the literature were screened independently by two researchers according to the inclusion and exclusion criteria, and then the full text was read for a second screening. Conflicting studies were re-evaluated by discussion or by seeking the advice of a third researcher. Two researchers independently extracted the data information of the final included literature using Excel 2016. Two researchers used Excel 2016 to independently extract the data information of the final included literature, including Study, Study type, Enrollment, Stage, Dose(Gy),Radiotherapy technique, Heart substructures, Cut-off value, Conclusion.

## 3 The correlation between heart dose and survival

The term RIHD was originally first described in the cardiac complications that arose in patients with breast cancer or lymphoma who received thoracic radiotherapy (13, 14). Radiation oncologists have long held the belief that RIHD was a delayed effect that primarily affected those who survived cancer for an extended period. However, this notion overlooked the significant impact that RIHD could have on patients with cancers that had a shorter survival rate, such as lung cancer, which has a 5-year survival rate of approximately 10%-20% (15). Following the RTOG 0617 study, an increasing number of researchers have turned their attention to the relationship between heart dose and survival in lung cancer patients receiving thoracic radiotherapy. On the one hand, lung cancer patients are typically diagnosed at an older age than breast cancer patients and tend to have more comorbidities, including cardiac complications. On the other hand, lung cancer patients receive higher radiation doses than breast cancer patients, which makes them less tolerant of heart irradiation, leading to earlier onset of cardiac adverse events. Thus, it is crucial to consider the radiation dose received by the heart during radiotherapy for lung cancer patients. **Table 1** provides a summary of the relevant studies published to date that investigate the relationship between radiation dose received by the whole heart and survival.

**Table 1:** Study on the correlation between heart dose and survival.

Study	Study type	Enrollment	Stage	Dose(gy)	Radiotherapy technique	Conclusion
Schytte et al. (16)	retrospective	328	I-III	60/66/80	3DCRT	MHD not associated with OS
Bradley et al. (11)	prospective	554	III	74 vs. 60	3DCRT/ IMRT/	V5, V30 associated with OS
Tucker et al. (21)	retrospective	468	IIIA/IIIB	63(60-76)	3DCRT/ IMRT/ PROTON	V5, MHD not associated with OS
Guberina et al. (17)	prospective	161	IIIA/IIIB	45 vs. 45+(20-26)	3DCRT	V5 not associated with survival
Dess et al. (22)	prospective	125	II/III	70(45-88)	IMRT/ 3DCRT	V5, V30, V50, MHD not associated with OS
Wang et al. (23)	prospective	127	III	74(70-90)	3DCRT	V5, V30, MHD not associated with OS
McWilliam et al. (24)	retrospective	1101	–	55	IMRT/ 3DCRT	V5, V30, MHD not associated with OS
Ning et al. (25)	prospective	201	I-IV	74(60-74)	PROTON/ IMRT	MHD not associated with OS
Chun et al. (12)	prospective	482	IIIA/IIIB	74 vs. 60	IMRT/ 3DCRT	V40 associated with OS
Vivekanandn et al. (26)	prospective	78	IIB-III	67.6(63-73)	3DCRT/ VMAT	V63–69 associated with OS
Ma et al. (27)	retrospective	141	III	66(60-76)	IMRT/ 3DCRT	V30, V35, V40, V45 not associated with OS
Stam et al. (28)	retrospective	469	IIA-IIIB	66	IMRT	V2 associated with OS
Speirs et al. (19)	retrospective	416	II-III	(50-84.9)	3D-CRT/ IMRT	V50 associated with OS
Contreras et al. (29)	retrospective	400	II-III	66(50-77.25)	3DCRT/ IMRT/ PROTON	V50 associated with OS
Yegya-Ramn et al. (30)	retrospective	140	IIA-IV	61.2(50.4-70.2)	3DCRT/ IMRT	MHD associated with OS; MHD not associated with V5, V30, V50
Xue et al. (31)	prospective	94	I-III	70(45–85.5)	3DCRT	V5, V30, V55, MHD not associated with OS
Atkins et al. (18)	retrospective	748	IIIB	64.0(54.9-66.0)	3DCRT/ IMRT	MHD associated with all-cause mortality

3DCRT, three dimensional conformal radiation therapy; IMRT, intensity modulated radiation therapy; PROTON, proton radiotherapy; MHD, mean heart dose; OS, overall survival.

Existing studies indicate a potential association between cardiac radiation dose and overall survival (OS) in lung cancer patients receiving radiotherapy, yet the conclusions remain inconsistent. Such variability largely reflects differences in patient population characteristics, the evolution of treatment techniques, and variations in follow-up duration. In early clinical cohorts, the high tumor-related mortality of lung cancer often obscured the long-term impact of radiation-induced cardiac injury, making it difficult to detect a significant correlation between cardiac dose and prognosis (16, 17). With the refinement of radiotherapy and chemotherapy, as well as the widespread adoption of consolidation immunotherapy, median OS has been markedly prolonged; consequently, the detrimental effect of cardiac irradiation on long-term survival has gradually become more evident, a phenomenon confirmed by large-scale studies in recent years (18).

Meanwhile, advances in radiotherapy are reshaping the relationship between cardiac dose and survival. During the 3D-CRT era, extensive irradiation field increased the volume of low-dose exposure, and as a result, low-dose and intermediate-dose parameters (V5, V30, V50) showed a trend toward correlation with OS in some studies—for example, Speirs et al. (19) reported a significant association between V50 and OS. With the widespread implementation of IMRT, however, greater conformity has led to the concentration of high-dose exposure in specific cardiac regions or substructures, such as the left anterior descending artery (LAD) and the left atrium. In this setting, high-dose parameters pertaining to these substructures appear to carry stronger prognostic value. Notably, Atkins et al. (20) reported that LAD V15≥10% was associated with a significantly increased risk of major adverse cardiac events and death (HR 1.58, 95% CI 1.09-2.29).

The development of proton therapy has substantially reduced exposure to mean heart dose (MHD). Nevertheless, results may be subject to bias due to stringent patient selection criteria. For instance, Tucker et al. (21) often selected high−risk cases with tumors situated close to the heart, a factor that may have contributed to an overestimation of the relationship between MHD and OS. Furthermore, the biological interpretations of different dosimetric parameters are not entirely consistent: MHD reflects only the global average and may underestimate the impact of focal high-risk exposure; intermediate and high-dose volume fractions (V30–V50) provide a better indication of risks such as cardiac fibrosis or large-vessel injury; and low-dose volume (V5) has been linked to systemic inflammatory responses or immunosuppression. It should also be emphasized that in the era of immunotherapy, prolonged survival has made delayed cardiotoxicity increasingly relevant, and accumulating evidence suggests that focal irradiation of critical substructures such as the LAD or atrium is associated with increased mortality risk (20).

## 4 The correlation between substructure heart dose and survival

According to some researchers, limiting the radiation dose to the heart as a whole organ is a crude method. As a result, scholars have divided the heart into several substructures to assess the radiation dose more accurately. **Table 2** provides a summary of relevant studies that explore the correlation between cardiac substructures dose and survival. In some studies, the substructures of the heart are defined based on its inherent basic structure, including the left and right atria, left and right ventricles, pericardium, coronary system, valves, and major blood vessels. Other studies have examined the correlation between self-defined special structures or regions and survival. In a retrospective study, McWilliam demonstrated that the radiation dose received by the bottom region of the heart was correlated with the survival of lung cancer patients undergoing radiotherapy (24). In another retrospective study, the same author defined a special region of the heart that included the right atrium, right coronary artery, and ascending aorta (32). The study revealed that patients with an equivalent dose in 2-Gy fractions (EQD2) greater than 23 Gy in this region had significantly shorter overall survival (OS) than those with an EQD2 of less than 23 Gy (EQD2 >23 Gy: 12 months, 95% CI: 10–14 months; EQD2 <23 Gy: 21 months, 95% CI: 17–23 months, P = 0.008) (32). However, recent studies have attempted to explore the correlation between established substructures of the heart and survival. For instance, Thor et al. utilized the RTOG 0617 database to establish a multifactorial survival prediction model (33). Cox multivariate analysis revealed that both the left atrium D45% (the minimum dose received by 45% of the volume) and the ventricular MOH5% (the average dose received by 5% of the volume) were independent prognostic factors for survival (33). In a study of 701 non-small cell lung cancer patients, Atkins et al. discovered that a coronary left anterior descending artery V15 ≥10% significantly increased the mortality of lung cancer patients (HR = 1.58, 95% CI: 1.09-2.29, P = 0.02)[33]. However, manually or automatically segmenting and delineating substructures of the heart remains a challenging and time-consuming task in routine radiotherapy planning (34–36). Therefore, the delineation of substructures of the heart has not yet been widely implemented in clinical practice.

**Table 2:** Study on the correlation between heart substructures dose and survival.

Study	Stage	Heart substructures	Cut-off value	HR (95%CI)	OS difference(months)
Xue et al. (31)	I-III	Pericardium V30	29%	1.019 (1.004-1.033)	13.3 vs. 35.8
		Pericardium V55	21%	1.030 (1.006-1.054)	13.3 vs. 30.0
McWilliam et al. (32)	–	Specific area (including right atrium, right coronary artery, ascending aorta) Dmax	19.5 Gy	1.010 (1.010-1.020)	12.0 vs. 21.0
McWilliam et al. (24)	–	Specific area (cardiac base) Specific doses	8.5Gy	1.250 (1.01-1.56)	NA
Ma et al. (27)	–	Pulmonary artery V40	80%	2.113 (1.014-4.936)	14.0 vs. 27.8
		Pulmonary artery V45	68%	2.660 (1.089-5.717)	13.5 vs. 37.9
		Pulmonary artery V50	45%	1.203 (0.062-2.056)	14.2 vs. 32.7
		Pulmonary artery V55	32%	1.489 (0.098-2.096)	10.9 vs. 41.8
Vivekanandan et al. (26)	IIB/III	Left atrial wall V63	2.2%	1.520 (1.070-2.170)	39.2 vs. 27.9
Thor et al. (33)	III	Atrium cordis D45%	44/30Gy	NA	NA
		Pericardium MOH55%	51/39Gy	NA	NA
		Ventricle MOH5%	56/41Gy	NA	NA
Atkins et al. (20)	II-III	Left anterior descending coronary artery V15	10%	1.580 (1.090-2.290)	NA
Olive et al. (37)	–	Ventricle Dmax	NA	1.020 (1.000-1.040)	NA
Stam et al. (38)	–	Left atrium D0%	NA	1.006 (NA)	NA
		Superior vena cava D90%	NA	1.025 (NA)	NA

**Table 2** shows that several studies have reported associations between radiation dose to specific cardiac substructures(such as the left anterior descending artery (LAD), left atrium, heart base, and pulmonary artery—and OS) whereas the MHD often failed to demonstrate statistical significance. This suggests that the effect of small, high risk cardiac substructures may be “diluted” when assessed using the MHD. At present, however, substantial heterogeneity exists among studies, including differing definitions and delineation methods for substructures, limited sample sizes, and variable results. For instance, McWilliam et al. (32) reported that the dose to the heart base was associated with OS, whereas certain chamber-based parameter (such as the mean dose to the right ventricle) did not demonstrate prognostic value, indicating that clinical significance may depend on both structural function and its spatial relationship to tumor location. Overall, when tumors are located in the left upper lobe or in the mediastinum adjacent to major vessels, particular attention should be paid to the coronary arteries and left atrium. Conversely, when the target volume is close to the pulmonary artery or heart base, limiting intermediate-dose to high-dose exposure in these regions becomes essential. In the future, the integration of automated segmentation and multicenter validation may enable the development of standardized substructure dose–survival models, which are expected to provide greater guidance than reliance on MHD alone.

## 5 Mechanisms of cardiac fibrosis in RIHD

The development of fibrosis is the primary damage caused by radiotherapy to the heart. Radiotherapy generates reactive oxygen species (ROS) by ionizing water molecules and damaging the mitochondrial respiratory chain, leading to ROS accumulation. The activation of enzymes such as NADPH oxidase and cyclooxygenase can also accelerate ROS accumulation. Meanwhile, radiation suppresses antioxidant enzymes, which impairs the ability of antioxidants to clear accumulated ROS, exacerbating oxidative stress and resulting in various chemical reactions in the body (39). Oxidative stress is closely associated with myocardial fibrosis. The release of proinflammatory factors, such as TNF-α, IL-1, and IL-11, as well as adhesion molecules, increases the number of fibroblasts (40). This leads to the formation of microthrombi and vascular occlusion, resulting in perfusion defects and focal ischemia, which exacerbate cardiomyocyte death and fibrosis (40). Myocardial fibrosis is primarily identified by the accumulation of collagen in the heart, which eventually replaces cardiomyocytes (41). Moreover, ROS and lipid peroxidation products can deactivate membrane-bound receptors and enzymes, resulting in increased tissue permeability, protein inactivation, and ultimately the destruction of cardiomyocyte membranes (41). Studies have shown that ROS and protein oxidation may impact the function of receptors, enzymes, and transport proteins (41). For instance, ROS can overactivated Ca^2+^-calmodulin-dependent protein kinase II, resulting in irregular excitation-contraction coupling, heart failure, and arrhythmia (42). Radiation-induced microvascular damage can cause elevated capillary permeability and the swift emergence and progression of protein-rich exudates, ultimately resulting in radiation-induced pericarditis (43). The accumulation of collagen in the interstitium and apex of the pericardium can also result in pericardial fibrosis.

The DNA double-strand breaks (DSBs) which is caused by the radiation and the ROS can activate I-κB kinase, which mediates I-κB degradation and releases NF-κB into the nucleus (44). NF-κB binds to the promoter regions of target genes, promoting the expression of NADPH oxidase and cyclooxygenase in target genes to result in further elevation of ROS levels (45). These ROS, in turn, continue to affect NF-κB, forming a positive feedback loop that speeds up the cardiac fibrosis. In addition, NF-κB also induces some pro-inflammatory factors such as TNF-α to increase the number of presenting cells.

Radiation-induced cardiac fibrosis frequently demonstrates overexpression of TGF-β, indicating that an elevated level of transforming growth factor may worsen RACD. Ionizing radiation damage can activate TGFβ through various pathways, including ROS generation, excessive inflammation activation, microvascular damage, platelet activation, and cellular aging and apoptosis (46). TGF-β can induce fibrosis through both the canonical and noncanonical signaling pathways. In the canonical pathway, TGF-β activates target genes, including type I collagen, type III collagen, CTGF, and α-smooth muscle actin, via Smad transcription factors (47). TGF-β can also exert its effects through non-Smad pathways, such as Rho/ROCK, which further enhance fibrosis. Simultaneously, TGF-β can strengthen the profibrotic signals mentioned earlier through ROS, resulting in the formation and accumulation of myofibroblasts and extracellular matrix and accelerating the onset and progression of fibrosis (48). The platelet-derived growth factor (PDGF) family of factors is another critical mediator of myocardial fibrosis. Research has revealed that the overexpression of cardiac PDGF-C and PDGF-D through transgenic technology leads to extensive cardiac fibrosis (49, 50).

## 6 Mechanisms of cardiac cell injury and death in RIHD

Radiation can cause various types of DNA damage, among which DNA double-strand breaks (DSBs) are the most severe. ROS and DSBs activate I-κB kinase, which mediates I-κB degradation and releases NF-κB into the nucleus (44). NF-κB binds to the promoter regions of target genes, inducing the expression of proinflammatory factors such as TNF-α, IL-1, IL-6, and IL-8, thereby regulating the inflammatory response (44). Simultaneously, NF-κB can enhance the adhesion ability of leukocytes by inducing the secretion of adhesion molecules (41). The infiltration of neutrophils can result in the additional release of various proinflammatory factors, worsening endothelial cell damage (41). Infiltrating monocytes can differentiate into activated macrophages, which struggle to degrade low-density lipoprotein oxidized by ROS, progressively transforming into foam cells, a process closely linked to the development of atherosclerosis (41). Furthermore, NF-κB promotes the expression of NADPH oxidase and cyclooxygenase in target genes, resulting in further elevation of ROS levels (45). These ROS, in turn, continue to affect NF-κB, forming a positive feedback loop that speeds up the progression of coronary artery disease and vascular damage (45).

Research has demonstrated that in the initial phases of radiotherapy, ROS and DNA damage repair (DDR) can boost NO by phosphorylating serine 1177 on endothelial nitric oxide synthase (eNOS) in human endothelial cells (51, 52). However, the interaction between ROS and NO results in reactive nitrogen species, which decreases the bioavailability of NO (53). Simultaneously, ROS stimulate the production of vasoconstrictive substances such as prostaglandins, which hinder vascular relaxation and eventually result in vascular stenosis (53). Moreover, radiotherapy can cause a reduction in myocardial capillaries, and increase the expression of von Willebrand factor in endothelial cells, leading to platelet adhesion and thrombus formation in blood vessels, worsening ischemia and hypoxia (54, 55). ROS and DNA damage signals trigger cell apoptosis through the Bcl-2/Bax protein family and the p53 protein, respectively (45, 54). The Bcl-2/Bax protein family can also cause cell apoptosis by changing mitochondrial permeability (56). Furthermore, radiotherapy can enhance the release of Ca^2+^ from the endoplasmic reticulum, resulting in an elevation of mitochondrial Ca^2+^ uptake (57). Calcium overload can ultimately lead to cell membrane swelling and the release of apoptotic factors (57).

## 7 The expression mechanism of micro-RNAs provides ideas for RIHD prediction

Several studies have suggested that micro-RNAs (miRNAs) are involved in the pathogenesis and progression of RIHD (58–60). Therefore, we believe that miRNAs can be used as an early molecular marker to predict heart damage. To begin with, exposure to ionizing radiation and other oxidative stress-inducing factors can lead to alterations in miRNA expression (58). Numerous investigations have demonstrated that miRNAs are implicated in the pathological processes related to cardiac radiation damage, such as oxidative stress, inflammation, endothelial dysfunction, hypertrophy, fibrosis, and subsequent heart failure (59, 60). Recently, miRNAs have also been found to be involved in the regulation of radiation-induced DNA damage (61). For instance, miRNA-21 has been shown to promote cell proliferation and anti-apoptosis (62). Csilla et al. reported that the expression of miRNA-21 in the myocardium was significantly increased following radiation, particularly in the left ventricle (63). On the other hand, miRNA-1 expression was down-regulated in irradiated animal models, consistent with changes in cardiac hypertrophy and heart failure, and altered in various cardiovascular diseases (59). Furthermore, changes in miRNA-34a expression have also been associated with heart injury, and a study has indicated that miRNA-34a expression was up-regulated after radiation exposure (64).

**Figure 1:**
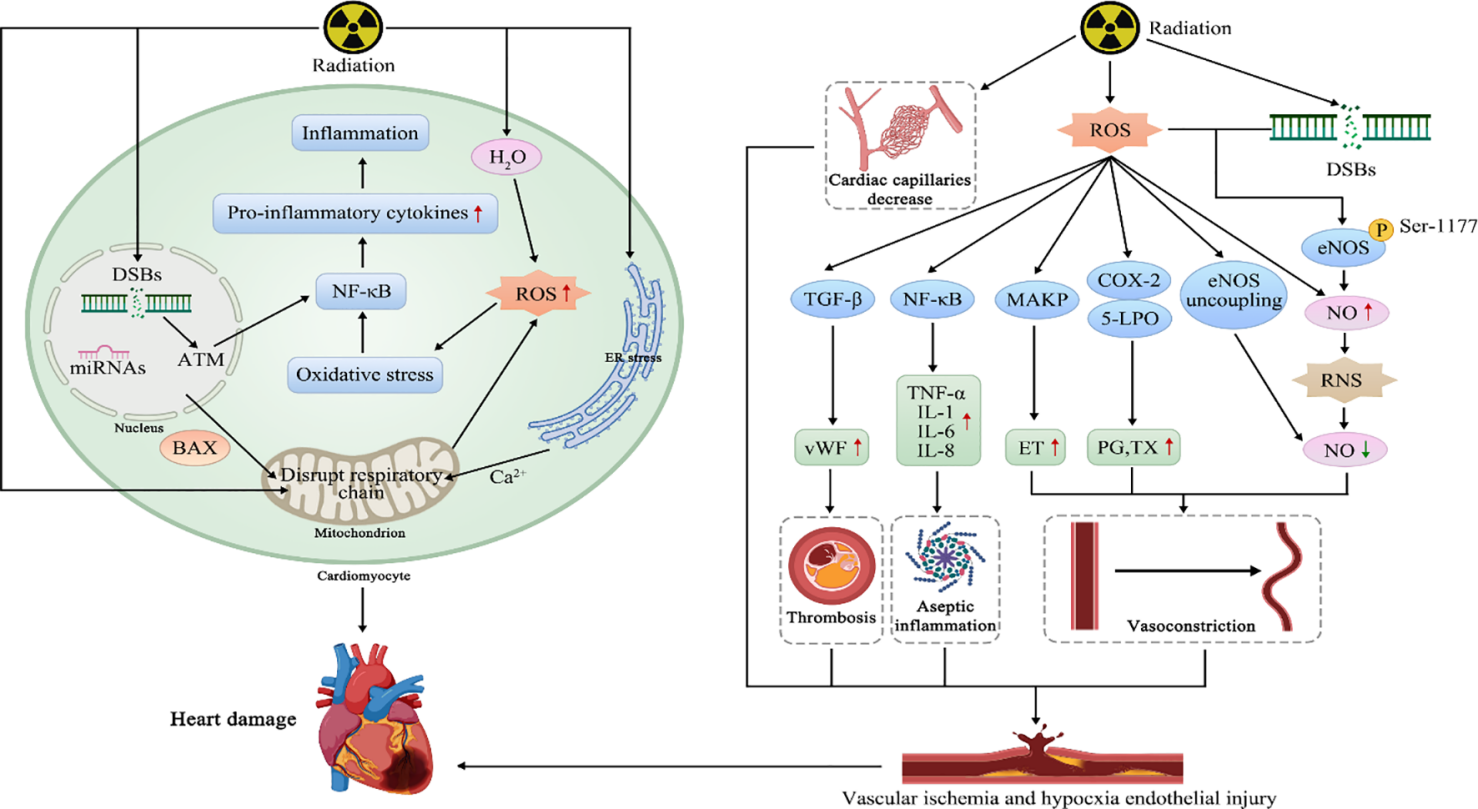
The mechanisms of the RIHD.

The above-described mechanisms are depicted in **Figure 1**.

## 8 Strategies for prevention and management of RIHD

### 8.1 Cardiac-sparing radiotherapy techniques

Preventive strategies mainly focus on reducing the cardiac irradiation dose. With conventional 3D conformal radiotherapy (3DCRT), considerable incidental exposure of the heart is common. Modern photon techniques such as intensity-modulated radiotherapy (IMRT) and volumetric modulated arc therapy (VMAT) provide more conformal dose distributions and significantly reduce heart volumes receiving intermediate to high doses (12).

Proton therapy has demonstrated superiority in reducing mean heart dose (MHD) and left anterior descending artery (LAD) exposure when compared to photon IMRT, as shown by randomized and dosimetric studies (65). Robust optimization and spread-out Bragg peak characteristics eliminate exit dose, leading to improved sparing of cardiac substructures.

Motion management techniques, including deep inspiration breath hold (DIBH), expiration breath hold, respiratory gating, and tumor tracking, have emerged as pivotal strategies to increase the distance between the tumor and critical cardiac structures (66). DIBH is widely adopted for left breast and mediastinal targets, and increasingly used in locally advanced lung cancer to reduce MHD.

Adaptive radiotherapy and MRI-guided RT enable daily plan adaptation and improved visualization of heart substructures. At the same time, AI-based automatic substructure delineation provides standardization and efficiency, overcoming the steep learning curve of manual segmentation (32, 67).

Collectively, these strategies highlight a paradigm shift from whole-heart dose limitation to substructure-specific constraints (eg, LAD V15 < 10% or pericardium V30 < 30%) with the aim of better predicting RIHD and survival outcomes (20, 66).

### 8.2 Management of established RIHD events

Once RIHD occurs, management resembles standard cardiology approaches. Arrhythmias may be treated with antiarrhythmic agents or pacemaker/ICD implantation. Heart failure is managed with beta-blockers, ACEIs/ARBs, diuretics, and guideline-directed therapy. Pericarditis responds to anti-inflammatory drugs and colchicine, while constrictive disease may require pericardiectomy. Coronary disease can be managed with percutaneous intervention or bypass grafts, and valvular damage may necessitate surgery (43, 55). In patients receiving immune checkpoint inhibitors, immune-related myocarditis requires corticosteroids and sometimes additional immunosuppressants (68). These treatments control symptoms and prevent progression, but do not reverse structural fibrotic changes induced by radiation.

### 8.3 Lifestyle and risk factor modification

Risk factor control is essential. Smoking cessation, strict management of hypertension, diabetes, and dyslipidemia, and the use of statins or aspirin in selected patients may reduce the burden of RIHD (69). Multidisciplinary “cardio-oncology” programs are increasingly important for high-risk patients undergoing thoracic RT (70).

## 9 Conclusion

Radiation-induced heart disease (RIHD) is an emerging determinant of survival in lung cancer patients receiving thoracic radiotherapy. Current evidence indicates that whole-heart mean dose alone is inadequate to describe clinically relevant risk, as the prognostic impact often arises from focal exposure of critical substructures such as the left anterior descending artery, left atrium, pulmonary artery, and heart base. This underscores the need to move from global dose metrics toward substructure-specific evaluation.

Recent advances (including IMRT, proton therapy, motion management, and adaptive radiotherapy) facilitate selective cardiac sparing, but heterogeneous delineation methods and limited prospective validation hinder the establishment of universal constraints. Future research should prioritize standardized segmentation, multicenter collaboration, and prospective dose-response modeling.

In the immunotherapy era, where patient survival is improving, refinement of cardiac-sparing strategies is essential to balance tumor control with long-term cardiovascular safety, ultimately optimizing both overall survival and quality of life in lung cancer patients.

## References

[1] Siegel RL Miller KD Jemal A. Cancer statistics, 2020. CA Cancer J Clin. (2020) 70:7–30. doi: 10.3322/caac.21590, PMID: 31912902

[2] Aupérin A Le Péchoux C Rolland E Curran WJ Furuse K Fournel P. Meta-analysis of concomitant versus sequential radiochemotherapy in locally advanced non-small-cell lung cancer. J Clin Oncol. (2010) 28:2181–90., PMID: 20351327 10.1200/JCO.2009.26.2543

[3] Ettinger DS Wood DE Aisner DL Akerley W Bauman JR Bharat A. Non-small cell lung cancer, version 3.2022, NCCN clinical practice guidelines in oncology. J Natl Compr Canc Netw. (2022) 20:497–530. doi: 10.6004/jnccn.2022.0025, PMID: 35545176

[4] Ahn JS Ahn YC Kim J-H Lee CG Cho EK Lee KC. Multinational randomized phase III trial with or without consolidation chemotherapy using docetaxel and cisplatin after concurrent chemoradiation in inoperable stage III non-small-cell lung cancer: KCSG-LU05-04. J Clin Oncol. (2015) 33:2660–6. doi: 10.1200/JCO.2014.60.0130, PMID: 26150444

[5] Salazar OM Slawson RG Poussin-Rosillo H Amin PP Sewchand W Strohl RA. A prospective randomized trial comparing once-a-week vs daily radiation therapy for locally-advanced, non-metastatic, lung cancer: a preliminary report. Int J Radiat Oncol Biol Phys. (1986) 12:779–87. doi: 10.1016/0360-3016(86)90036-2, PMID: 3519551

[6] Bradley JD Moughan J Graham MV Byhardt R Govindan R Fowler J. A phase I/II radiation dose escalation study with concurrent chemotherapy for patients with inoperable stages I to III non-small-cell lung cancer: phase I results of RTOG 0117. Int J Radiat Oncol Biol Phys. (2010) 77:367–72. doi: 10.1016/j.ijrobp.2009.04.029, PMID: 20457350 PMC2869096

[7] Schild SE McGinnis WL Graham D Hillman S Fitch TR Northfelt D. Results of a Phase I trial of concurrent chemotherapy and escalating doses of radiation for unresectable non-small-cell lung cancer. Int J Radiat Oncol Biol Phys. (2006) 65:1106–11. doi: 10.1016/j.ijrobp.2006.02.046, PMID: 16730134

[8] Socinski MA Blackstock AW Bogart JA Wang X Munley M Rosenman J. Randomized phase II trial of induction chemotherapy followed by concurrent chemotherapy and dose-escalated thoracic conformal radiotherapy (74 Gy) in stage III non-small-cell lung cancer: CALGB 30105. J Clin Oncol. (2008) 26:2457–63. doi: 10.1200/JCO.2007.14.7371, PMID: 18487565

[9] Stinchcombe TE Lee CB Moore DT Rivera MP Halle J Limentani S. Long-term follow-up of a phase I/II trial of dose escalating three-dimensional conformal thoracic radiation therapy with induction and concurrent carboplatin and paclitaxel in unresectable stage IIIA/B non-small cell lung cancer. J Thorac Oncol. (2008) 3:1279–85. doi: 10.1097/JTO.0b013e31818b1971, PMID: 18978563

[10] Machtay M Bae K Movsas B Paulus R Gore EM Komaki R. Higher biologically effective dose of radiotherapy is associated with improved outcomes for locally advanced non-small cell lung carcinoma treated with chemoradiation: an analysis of the Radiation Therapy Oncology Group. Int J Radiat Oncol Biol Phys. (2012) 82:425–34. doi: 10.1016/j.ijrobp.2010.09.004, PMID: 20980108 PMC5764542

[11] Bradley JD Paulus R Komaki R Masters G Blumenschein G Schild S. Standard-dose versus high-dose conformal radiotherapy with concurrent and consolidation carboplatin plus paclitaxel with or without cetuximab for patients with stage IIIA or IIIB non-small-cell lung cancer (RTOG 0617): a randomised, two-by-two factorial phase 3 study. Lancet Oncol. (2015) 16:187–99. doi: 10.1016/S1470-2045(14)71207-0, PMID: 25601342 PMC4419359

[12] Chun SG Hu C Choy H Komaki RU Timmerman RD Schild SE. Impact of intensity-modulated radiation therapy technique for locally advanced non-small-cell lung cancer: A secondary analysis of the NRG oncology RTOG 0617 randomized clinical trial. J Clin Oncol. (2017) 35:56–62. doi: 10.1200/JCO.2016.69.1378, PMID: 28034064 PMC5455690

[13] Darby SC Ewertz M McGale P Bennet AM Blom-Goldman U Brønnum D. Risk of ischemic heart disease in women after radiotherapy for breast cancer. N Engl J Med. (2013) 368:987–98. doi: 10.1056/NEJMoa1209825, PMID: 23484825

[14] Haddy N Diallo S El-Fayech C Schwartz B Pein F Hawkins M. Cardiac diseases following childhood cancer treatment: cohort study. Circulation. (2016) 133:31–8. doi: 10.1161/CIRCULATIONAHA.115.016686, PMID: 26487757

[15] Bray F Ferlay J Soerjomataram I Siegel RL Torre LA Jemal A. Global cancer statistics 2018: GLOBOCAN estimates of incidence and mortality worldwide for 36 cancers in 185 countries. CA Cancer J Clin. (2018) 68:394–424. doi: 10.3322/caac.21492, PMID: 30207593

[16] Schytte T Hansen O Stolberg-Rohr T Brink C. Cardiac toxicity and radiation dose to the heart in definitive treated non-small cell lung cancer. Acta Oncol. (2010) 49:1058–60. doi: 10.3109/0284186X.2010.504736, PMID: 20831496

[17] Guberina M Eberhardt W Stuschke M Gauler T Heinzelmann F Cheufou D. Heart dose exposure as prognostic marker after radiotherapy for resectable stage IIIA/B non-small-cell lung cancer: secondary analysis of a randomized trial. Ann Oncol. (2017) 28:1084–9. doi: 10.1093/annonc/mdx069, PMID: 28453703

[18] Atkins KM Rawal B Chaunzwa TL Lamba N Bitterman DS Williams CL. Cardiac radiation dose, cardiac disease, and mortality in patients with lung cancer. J Am Coll Cardiol. (2019) 73:2976–87. doi: 10.1016/j.jacc.2019.03.500, PMID: 31196455

[19] Speirs CK DeWees TA Rehman S Molotievschi A Velez MA Mullen D. Heart dose is an independent dosimetric predictor of overall survival in locally advanced non-small cell lung cancer. J Thorac Oncol. (2017) 12:293–301. doi: 10.1016/j.jtho.2016.09.134, PMID: 27743888

[20] Atkins KM Chaunzwa TL Lamba N Bitterman DS Rawal B Bredfeldt J. Association of left anterior descending coronary artery radiation dose with major adverse cardiac events and mortality in patients with non-small cell lung cancer. JAMA Oncol. (2021) 7:206–19. doi: 10.1001/jamaoncol.2020.6332, PMID: 33331883 PMC7747040

[21] Tucker SL Liu A Gomez D Tang LL Allen P Yang J. Impact of heart and lung dose on early survival in patients with non-small cell lung cancer treated with chemoradiation. Radiother Oncol. (2016) 119:495–500. doi: 10.1016/j.radonc.2016.04.025, PMID: 27189523

[22] Dess RT Liu A Gomez D Tang LL Allen P Yang J. Cardiac events after radiation therapy: combined analysis of prospective multicenter trials for locally advanced non-small-cell lung cancer. J Clin Oncol. (2017) 35:1395–402. doi: 10.1200/JCO.2016.71.6142, PMID: 28301264 PMC5455464

[23] Wang K Eblan MJ Deal AM Lipner M Zagar TM Wang Y. Cardiac toxicity after radiotherapy for stage III non-small-cell lung cancer: pooled analysis of dose-escalation trials delivering 70 to 90 gy. J Clin Oncol. (2017) 35:1387–94. doi: 10.1200/JCO.2016.70.0229, PMID: 28113017 PMC5455462

[24] McWilliam A Kennedy J Hodgson C Vasquez Osorio E Faivre-Finn C van Herk M. Radiation dose to heart base linked with poorer survival in lung cancer patients. Eur J Cancer. (2017) 85:106–13. doi: 10.1016/j.ejca.2017.07.053, PMID: 28898766

[25] Ning MS Tang L Gomez DR Xu T Luo Y Huo J. Incidence and predictors of pericardial effusion after chemoradiation therapy for locally advanced non-small cell lung cancer. Int J Radiat Oncol Biol Phys. (2017) 99:70–9. doi: 10.1016/j.ijrobp.2017.05.022, PMID: 28816165 PMC5667664

[26] Vivekanandan S Landau DB Counsell N Warren DR Khwanda A Rosen SD. The impact of cardiac radiation dosimetry on survival after radiation therapy for non-small cell lung cancer. Int J Radiat Oncol Biol Phys. (2017) 99:51–60. doi: 10.1016/j.ijrobp.2017.04.026, PMID: 28816160 PMC5554783

[27] Ma J-T Sun L Sun X Xiong Z-C Liu Y Zhang S-L. Is pulmonary artery a dose-limiting organ at risk in non-small cell lung cancer patients treated with definitive radiotherapy? Radiat Oncol. (2017) 12:34. doi: 10.1186/s13014-017-0772-5, PMID: 28143532 PMC5286829

[28] Stam B van der Bijl E van Diessen J Rossi MMG Tijhuis A Belderbos JSA. Heart dose associated with overall survival in locally advanced NSCLC patients treated with hypofractionated chemoradiotherapy. Radiother Oncol. (2017) 125:62–5. doi: 10.1016/j.radonc.2017.09.004, PMID: 28939179

[29] Contreras JA Lin AJ Weiner A Speirs C Samson P Mullen D. Cardiac dose is associated with immunosuppression and poor survival in locally advanced non-small cell lung cancer. Radiother Oncol. (2018) 128:498–504. doi: 10.1016/j.radonc.2018.05.017, PMID: 29859754

[30] Yegya-Raman N Wang K Kim S Reyhan M Deek MP Sayan M. Dosimetric predictors of symptomatic cardiac events after conventional-dose chemoradiation therapy for inoperable NSCLC. J Thorac Oncol. (2018) 13:1508–18. doi: 10.1016/j.jtho.2018.05.028, PMID: 29883836 PMC10905612

[31] Xue J Han C Jackson A Hu C Yao H Wang W. Doses of radiation to the pericardium, instead of heart, are significant for survival in patients with non-small cell lung cancer. Radiother Oncol. (2019) 133:213–9. doi: 10.1016/j.radonc.2018.10.029, PMID: 30416046 PMC6445767

[32] McWilliam A Khalifa J Vasquez Osorio E Banfill K Abravan A Faivre-Finn C. Novel methodology to investigate the effect of radiation dose to heart substructures on overall survival. Int J Radiat Oncol Biol Phys. (2020) 108:1073–81. doi: 10.1016/j.ijrobp.2020.06.031, PMID: 32585334

[33] Thor M Deasy JO Hu C Gore E Bar-Ad V Robinson C. Modeling the impact of cardiopulmonary irradiation on overall survival in NRG oncology trial RTOG 0617. Clin Cancer Res. (2020) 26:4643–50. doi: 10.1158/1078-0432.CCR-19-2627, PMID: 32398326 PMC7877447

[34] Zhu J Zhang J Qiu B Liu Y Liu X Chen L. Comparison of the automatic segmentation of multiple organs at risk in CT images of lung cancer between deep convolutional neural network-based and atlas-based techniques. Acta Oncol. (2019) 58:257–64. doi: 10.1080/0284186X.2018.1529421, PMID: 30398090

[35] Finnegan R Dowling J Koh E-S Tang S Otton J Delaney G. Feasibility of multi-atlas cardiac segmentation from thoracic planning CT in a probabilistic framework. Phys Med Biol. (2019) 64:085006. doi: 10.1088/1361-6560/ab0ea6, PMID: 30856618

[36] Zhou R Liao Z Pan T Milgrom SA Pinnix CC Shi A. Cardiac atlas development and validation for automatic segmentation of cardiac substructures. Radiother Oncol. (2017) 122:66–71. doi: 10.1016/j.radonc.2016.11.016, PMID: 27939201 PMC5292289

[37] Wong OY Yau V Kang J Glick D Lindsay P Le LW. Survival impact of cardiac dose following lung stereotactic body radiotherapy. Clin Lung Cancer. (2018) 19:e241–6. doi: 10.1016/j.cllc.2017.08.002, PMID: 28941961

[38] Stam B Peulen H Guckenberger M Mantel F Hope A Werner-Wasik M. Dose to heart substructures is associated with non-cancer death after SBRT in stage I-II NSCLC patients. Radiother Oncol. (2017) 123:370–5. doi: 10.1016/j.radonc.2017.04.017, PMID: 28476219

[39] Azzam EI Jay-Gerin J-P Pain D. Ionizing radiation-induced metabolic oxidative stress and prolonged cell injury. Cancer Lett. (2012) 327:48–60. doi: 10.1016/j.canlet.2011.12.012, PMID: 22182453 PMC3980444

[40] Wang B Wei J Meng L Wang H Qu C Chen X. Advances in pathogenic mechanisms and management of radiation-induced fibrosis. BioMed Pharmacother. (2020) 121:109560. doi: 10.1016/j.biopha.2019.109560, PMID: 31739160

[41] Ping Z Peng Y Lang H Xinyong C Zhiyi Z Xiaocheng W. Oxidative stress in radiation-induced cardiotoxicity. Oxid Med Cell Longev. (2020) 2020:3579143. doi: 10.1155/2020/3579143, PMID: 32190171 PMC7071808

[42] Luczak ED Anderson ME. CaMKII oxidative activation and the pathogenesis of cardiac disease. J Mol Cell Cardiol. (2014) 73:112–6. doi: 10.1016/j.yjmcc.2014.02.004, PMID: 24530899 PMC4048820

[43] Donnellan E Phelan D McCarthy CP Collier P Desai M Griffin B. Radiation-induced heart disease: A practical guide to diagnosis and management. Cleve Clin J Med. (2016) 83:914–22. doi: 10.3949/ccjm.83a.15104, PMID: 27938516

[44] Morgan MJ Liu Z. Crosstalk of reactive oxygen species and NF-κB signaling. Cell Res. (2011) 21:103–15. doi: 10.1038/cr.2010.178, PMID: 21187859 PMC3193400

[45] Ahamed J Laurence J. Role of platelet-derived transforming growth factor-β1 and reactive oxygen species in radiation-induced organ fibrosis. Antioxid Redox Signal. (2017) 27:977–88. doi: 10.1089/ars.2017.7064, PMID: 28562065 PMC5649128

[46] Baselet B Sonveaux P Baatout S Aerts A. Pathological effects of ionizing radiation: endothelial activation and dysfunction. Cell Mol Life Sci. (2019) 76:699–728. doi: 10.1007/s00018-018-2956-z, PMID: 30377700 PMC6514067

[47] Lee C-L Moding EJ Cuneo KC Li Y Sullivan JM Mao L. p53 functions in endothelial cells to prevent radiation-induced myocardial injury in mice. Sci Signal. (2012) 5:ra52. doi: 10.1126/scisignal.2002918, PMID: 22827996 PMC3533440

[48] Boerma M Kruse JJCM van Loenen M Klein HR Bart CI Zurcher C. Increased Deposition of vonWillebrand Factor in the Rat Heart after Local IonizingIrradiation. Strahlenther Onkol. (2004) 180:109–16. doi: 10.1007/s00066-004-1138-0, PMID: 14762664

[49] Sridharan V Aykin-Burns N Tripathi P Krager KJ Sharma SK Moros EG. Radiation-induced alterations in mitochondria of the rat heart. Radiat Res. (2014) 181:324–34. doi: 10.1667/RR13452.1, PMID: 24568130 PMC4029615

[50] Livingston K Schlaak RA Puckett LL Bergom C. The role of mitochondrial dysfunction in radiation-induced heart disease: from bench to bedside. Front Cardiovasc Med. (2020) 7:20. doi: 10.3389/fcvm.2020.00020, PMID: 32154269 PMC7047199

[51] Farhood B Khodamoradi E Hoseini-Ghahfarokhi M Motevaseli E Mirtavoos-Mahyari H Eleojo Musa A. TGF-β in radiotherapy: Mechanisms of tumor resistance and normal tissues injury. Pharmacol Res. (2020) 155:104745. doi: 10.1016/j.phrs.2020.104745, PMID: 32145401

[52] Zhang YE. Non-smad signaling pathways of the TGF-β Family. Cold Spring Harb Perspect Biol. (2017) 9:a022129. doi: 10.1101/cshperspect.a022129, PMID: 27864313 PMC5287080

[53] Pontén A Li X Thorén P Aase K Sjöblom T Ostman A. Transgenic overexpression of platelet-derived growth factor-C in the mouse heart induces cardiac fibrosis, hypertrophy, and dilated cardiomyopathy. Am J Pathol. (2003) 163:673–82. doi: 10.1016/S0002-9440(10)63694-2, PMID: 12875986 PMC1868211

[54] Pontén A Folestad EB Pietras K Eriksson U. Platelet-derived growth factor D induces cardiac fibrosis and proliferation of vascular smooth muscle cells in heart-specific transgenic mice. Circ Res. (2005) 97:1036–45. doi: 10.1161/01.RES.0000190590.31545.d4, PMID: 16224065

[55] Wang H Wei J Zheng Q Meng L Xin Y Yin X. Radiation-induced heart disease: a review of classification, mechanism and prevention. Int J Biol Sci. (2019) 15:2128–38. doi: 10.7150/ijbs.35460, PMID: 31592122 PMC6775290

[56] Sakata K Kondo T Mizuno N Shoji M Yasui H Yamamori T. Roles of ROS and PKC-βII in ionizing radiation-induced eNOS activation in human vascular endothelial cells. Vascul Pharmacol. (2015) 70:55–65. doi: 10.1016/j.vph.2015.03.016, PMID: 25869503

[57] Nagane M Kuppusamy ML An J Mast JM Gogna R Yasui H. Ataxia-telangiectasia mutated (ATM) kinase regulates eNOS expression and modulates radiosensitivity in endothelial cells exposed to ionizing radiation. Radiat Res. (2018) 189:519–28. doi: 10.1667/RR14781.1, PMID: 29474156

[58] Simone NL Soule BP Ly D Saleh AD Savage JE Degraff W. Ionizing radiation-induced oxidative stress alters miRNA expression. PloS One. (2009) 4:e6377. doi: 10.1371/journal.pone.0006377, PMID: 19633716 PMC2712071

[59] Kura B Yin C Frimmel K Krizak J Okruhlicova L Kukreja RC. Changes of microRNA-1, -15b and -21 levels in irradiated rat hearts after treatment with potentially radioprotective drugs. Physiol Res. (2016) 65 Suppl:1, S129–137. doi: 10.33549/physiolres, PMID: 27643935

[60] Slezak J Kura B Ravingerová T Tribulova N Okruhlicova L Barancik M. Mechanisms of cardiac radiation injury and potential preventive approaches. Can J Physiol Pharmacol. (2015) 93:737–53. doi: 10.1139/cjpp-2015-0006, PMID: 26030720

[61] Wang Y Scheiber MN Neumann C Calin GA Zhou D. MicroRNA regulation of ionizing radiation-induced premature senescence. Int J Radiat Oncol Biol Phys. (2011) 81:839–48. doi: 10.1016/j.ijrobp.2010.09.048, PMID: 21093163 PMC3056910

[62] Zhu H Fan G-C. Role of microRNAs in the reperfused myocardium towards post-infarct remodelling. Cardiovasc Res. (2012) 94:284–92. doi: 10.1093/cvr/cvr291, PMID: 22038740 PMC3331611

[63] Viczenczova C Kura B Egan Benova T Yin C Kukreja RC Slezak J. Irradiation-induced cardiac connexin-43 and miR-21 responses are hampered by treatment with atorvastatin and aspirin. Int J Mol Sci. (2018) 19:1128. doi: 10.3390/ijms19041128, PMID: 29642568 PMC5979305

[64] Hu Y Xia W Hou M. Macrophage migration inhibitory factor serves a pivotal role in the regulation of radiation-induced cardiac senescencethrough rebalancing the microRNA-34a/sirtuin 1 signaling pathway. Int J Mol Med. (2018) 42:2849–58. doi: 10.3892/ijmm.2018.3838, PMID: 30226567

[65] Liao Z Lee JJ Komaki R Gomez DR O’Reilly MS Fossella FV. Bayesian adaptive randomization trial of passive scattering proton therapy and intensity-modulated photon radiotherapy for locally advanced non-small-cell lung cancer. J Clin Oncol. (2018) 36:1813–22. doi: 10.1200/JCO.2017.74.0720, PMID: 29293386 PMC6008104

[66] Chan MF Parikh D Shi C. Narrative review: cardiotoxicities and cardiac-sparing techniques in radiotherapy. Technol Cancer Res Treat. (2024) 23:15330338241301211. doi: 10.1177/15330338241301211, PMID: 39636079 PMC11622324

[67] Chen C Qin C Qiu H Tarroni G Duan J Bai W. Deep learning for cardiac image segmentation: A review. Front Cardiovasc Med. (2020) 7:25. doi: 10.3389/fcvm.2020.00025, PMID: 32195270 PMC7066212

[68] Salem J-E Manouchehri A Moey M Lebrun-Vignes B Bastarache L Pariente A. Cardiovascular toxicities associated with immune checkpoint inhibitors: an observational, retrospective, pharmacovigilance study. Lancet Oncol. (2018) 19:1579–89. doi: 10.1016/S1470-2045(18)30608-9, PMID: 30442497 PMC6287923

[69] Yusuf S Joseph P Rangarajan S Islam S Mente A Hystad P. Modifiable risk factors, cardiovascular disease, and mortality in 155 722 individuals from 21 high-income, middle-income, and low-income countries (PURE): a prospective cohort study. Lancet. (2020) 395:795–808. doi: 10.1016/S0140-6736(19)32008-2, PMID: 31492503 PMC8006904

[70] Lyon AR Dent S Stanway S Earl H Brezden-Masley C Cohen-Solal A. Baseline cardiovascular risk assessment in cancer patients scheduled to receive cardiotoxic cancer therapies: a position statement and new risk assessment tools from the Cardio-Oncology Study Group of the Heart Failure Association of the European Society of Cardiology in collaboration with the International Cardio-Oncology Society. Eur J Heart Fail. (2020) 22:1945–60. doi: 10.1002/ejhf.1920, PMID: 32463967 PMC8019326

